# AAV2-mediated and hypoxia response element-directed expression of bFGF in neural stem cells showed therapeutic effects on spinal cord injury in rats

**DOI:** 10.1038/s41419-021-03546-6

**Published:** 2021-03-15

**Authors:** Sipin Zhu, Yibo Ying, Jiahui Ye, Min Chen, Qiuji Wu, Haicheng Dou, Wenfei Ni, Huazi Xu, Jiake Xu

**Affiliations:** 1 Department of Orthopaedics, The Second Affiliated Hospital and Yuying Children’s Hospital of Wenzhou Medical University, Wenzhou, 325000 Zhejiang China; 2 The Second School of Medicine, Wenzhou Medical University, 325027 Wenzhou, China; 3 School of Biomedical Sciences, The University of Western Australia, Perth, WA 6009 Australia

**Keywords:** Cell death in the nervous system, Neural stem cells

## Abstract

Neural stem cell (NSCs) transplantation has been one of the hot topics in the repair of spinal cord injury (SCI). Fibroblast growth factor (FGF) is considered a promising nerve injury therapy after SCI. However, owing to a hostile hypoxia condition in SCI, there remains a challenging issue in implementing these tactics to repair SCI. In this report, we used adeno-associated virus 2 (AAV2), a prototype AAV used in clinical trials for human neuron disorders, basic FGF (bFGF) gene under the regulation of hypoxia response element (HRE) was constructed and transduced into NSCs to yield AAV2-5HRE-bFGF-NSCs. Our results showed that its treatment yielded temporally increased expression of bFGF in SCI, and improved scores of functional recovery after SCI compared to vehicle control (AAV2-5HRE-NSCs) based on the analyses of the inclined plane test, Basso–Beattie–Bresnahan (BBB) scale and footprint analysis. Mechanistic studies showed that AAV2-5HRE-bFGF-NSCs treatment increased the expression of neuron-specific neuronal nuclei protein (NeuN), neuromodulin GAP43, and neurofilament protein NF200 while decreased the expression of glial fibrillary acidic protein (GFAP) as compared to the control group. Further, the expressions of autophagy-associated proteins LC3-II and Beclin 1 were decreased, whereas the expression of P62 protein was increased in AAV2-5HRE-bFGF-NSCs treatment group. Taken together, our data indicate that AAV2-5HRE-bFGF-NSCs treatment improved the recovery of SCI rats, which is accompanied by evidence of nerve regeneration, and inhibition of SCI-induced glial scar formation and cell autophagy. Thus, this study represents a step forward towards the potential use of AAV2-5HRE-bFGF-NSCs for future clinical trials of SCI repair.

## Introduction

Spinal cord injury (SCI) causes abnormal changes of the structure and function of the spinal cord, leading to high morbidity and mortality with huge burden to individuals, families, and society, and is a worldwide challenging medical problem^1–3^. Due to the non-reproducibility of central neurons, physical therapy, drug therapy, rehabilitation therapy, and other treatments have little significant efficacy on the recovery of the functions of injured nerves^4^. The self-renewal and multidirectional differentiation ability of neural stem cell (NSCs) emerge to play a role in the site of SCI, paving the way for the application of NSCs to treat SCI^5–7^. However, this approach is facing a major hurdle, as early microcirculation disorder in SCI caused by the damaged nerve fibers, local edema, ischemia, and hypoxia can lead to anaerobic metabolic acidosis and free radical reaction, as well as a series of secondary injury^8–10^. In addition, NSCs virtually are unable to tolerate hypoxia environment of SCI, which is often accompanied with pathogenic autophagy of neuron cells^11^, causing further deterioration of SCI.

Autophagy refers to the process in which cells wrap their damaged organelles or unwanted proteins into a double membrane structure and direct them to lysosomes for degradation^12^. It participates in the regulation of many diseases, including SCI. As a dynamic phenomenon in the biological procedure, autophagy is important in the growth and development of cell, and differentiation and survival of NSCs^13,14^. When autophagy becomes excessive, the abnormal proteins in the cells are congregated to a degree that causes impairment of the normal activities of the cells. Autophagy is induced by a variety of stress stimuli, which include hypoxia, endoplasmic reticulum stress, DNA damage, redox stress, and mitochondrial damage^15–17^. Excessive autophagy also hinders the stable growth and effective repair of nerve cells after SCI^18^. Autophagy was also involved in cell death especially in neurons and spinal cord glia cells after SCI^19–21^. Thus, current therapeutic interventions for SCI using neurotrophic factors aim to reduce the damage caused by excessive autophagy of spinal cord cells, and protect the secondary injury, such as exposure to free radicals and undue misfolded proteins.

Two classical pathways exist to regulate autophagy pathways, including mTOR pathway mediated by PI3k-ATK and phosphatase and tensin homolog (PTEN) phosphatase, and Beclin 1-Vps-34 complex pathway^22,23^. When the Beclin 1 and membrane-associated form of microtubule-related protein light chain 3 (LC3-II) are over-expressed, the excessive stimulation of autophagy occurs and results in the death of cells. LC3-II is located on autophagic vesicles in mammalian cells and has been used as a marker to track autophagy^24,25^. P62 is involved in binding to and degrading polyubiquitin-mediated proteins and exerts an important role in the signaling of autophagy^26^.

Basic fibroblast growth factor (bFGF) plays a significant role in the growth and development of the nervous system and is considered a candidate molecule for SCI repair^14^. Researchers have tried to deploy bFGF into SCI site and found the therapeutic effect was not obvious by these common deliver approaches^27,28^. Further, combined bFGF with stem cell therapy for delivery has been used, but its repair effect on the site of SCI was limited, and the recovery outcome of spinal cord was also not obvious^29,30^. The major hurdle of these approaches may lie in the fact that nerve cells were unable to survive efficiently under hostile hypoxia conditions, and to self-regulate to overcome the adverse effects^31–33^. Effective SCI repair may require cells to overcome hypoxia microenvironment and autophagy of cell death^34–36^. Therefore, how to turn the adverse hypoxia microenvironment into a favorable strategy to regulate bFGF expression and improve the survival of NSCs is particularly interesting.

In this study, we aim to employ hypoxia-response element (HRE) to mediate the expression human bFGF with adeno-associated virus 2 (AAV2) as a vector, which has a neural cell tropism and could potentially pave the way for future clinical trials^37,38^. To this end, AAV2-5HRE-bFGF-NSCs were generated and transplanted to the site of SCI. The effects of AAV2-5HRE-bFGF-NSCs on SCI rats were assessed using an array of analyses such as inclined plane test, Basso–Beattie–Bresnahan (BBB) scale, the footprint analysis, and video recording images. Further, the expressions of neuron-specific molecules and autophagy-related proteins such as LC3-II, Beclin 1, and P62 were examined. Our data revealed that AAV2-5HRE-bFGF-NSCs have therapeutic effect on SCI in rats via modulating hypoxia niche, and this preclinical study may pave the way for future clinical trials for the use of AAV2-5HRE-bFGF-NSCs for SCI repair.

## Materials and methods

### Reagents and antibodies

NSCs culture medium was provided by Chi Scientific (Jiangsu, China). Antibodies to HIF-1α (ab1), bFGF (ab208687), LC3-II (ab192890), Beclin 1 (ab217179), P62 (ab109012), NeuN (ab177487), GAP43 (ab75810), GFAP (ab33922), and NF200 (ab4680) were supplied by Abcam (Cambridge, Britain). Goat anti-rabbit IgG- conjugated with Alexa Fluor594 (red, ab150080) or Alexa-Fluor488 (green, ab150077) was from Abcam (Cambridge, Britain). Goat anti-mouse IgG-conjugated with Alexa Fluor594 (red, ab150116) or Alexa-Fluor488 (green, ab150113) was from Abcam (Cambridge, Britain). Goat anti-chicken IgY-conjugated with Alexa Fluor594 (red, ab150172) was from Abcam (Cambridge, Britain). An enhanced chemiluminescence (ECL) kit was provided by Bio-Rad (Hercules, CA, USA). DAPI (4′,6-diamidino-2-phenylindole), a fluorescent agent for cell nuclear staining was from Sigma-Aldrich (St. Louis, MO, USA). Rapamycin (RAPA) autophagy inducer was obtained from Cell Signaling Technology (Danvers, MA, United States), and 3-Methyladenine (3-MA) from Sigma-Aldrich (St. Louis, MO, USA).

### Isolation of embryonic NSCs from rats and cell culture

All animal experiments including rat NSCs used in this study were approved by the Animal Care and Use Committee of Wenzhou Medical College (wydw2014-0074) and conducted according to the guidelines of the Care and Use of Laboratory Animals set by the National Institutes of Health. The embryos of Sprague-Dawley rats aged from 14 to 17 days gestation were used to isolate embryonic NSCs according to our previous protocol^39^. NSCs were placed on a cell culture plate cultured in Dulbecco’s modified Eagle’s medium with bFGF (20 ng/ml) and epidermal growth factor (EGF, 20 ng/ml) in a 5% CO_2_ cell incubator. NSCs were left continue to divide to form a single ball of nerve cells and stay to proliferate until there were ~150 balls of nerve cells in the cell culture plate. The cultured NSCs were verified with the expression of nestin protein by immunofluorescence staining. NSCs were treated with RAPA (100 nM) or 3-MA (5 mM) for 12 h to examine their effects on autophagic pathways. All experiments were carried out in triplicate.

### Generation of NSCs transduced with AAV2-mediated and HRE-directed expression of bFGF gene

AAV2-5HRE-bFGF or AAV2-5HRE vectors were produced using pAOV001 pAAV-CAG-MCS backbone plasmid in the cloning site of Mlu I and Hind III with an outsourcing service from Obio (Shanghai, China). The identities of AAV2-5HRE-bFGF or AAV2-5HRE vectors were verified by DNA sequencing. Note that 5HRE represents five repeats of HRE DNA sequence, which were placed upstream of a cytomegalovirus (CMV) minimum promoter. The production of stocks of AAV2 virus particles was performed in the facility with an outsourcing service from Obio (Shanghai, China). In brief, AAV-2-5HRE-bFGF or AAV2-5HRE plasmids and the helper plasmid pAAV-RC were co-transfected and propagated in HEK293 cells. The virus particles were purified using caesium chloride density gradient followed by dialysis. The titers of the viruses were then verified by quantitative polymerase chain reaction (qPCR). It was estimated that stocks of AAV2-5HRE-bFGF and AAV2-5HRE have a titer of 2.15 × 10^13^ and 8.82 × 10^12^/ml virus particles; respectively, which were then employed for subsequent NSCs transduction experiments. In brief, NSCs were transduced with 1 × 10^3^ multiplicity of infection (MOI) of AAV2-5HRE-bFGF or AAV2-5HRE virus particles alone, which were repeatedly estimated to achieve a 95% transduction rate. Transduced cells were then cultivated in normoxia culture condition or in hypoxic box (<1% O_2_) for the hypoxia induced expression, 6 h or more. The culture supernatant for protein concentration was detected using bFGF enzyme-linked immunosorbent assay (ELISA) kit (Institute of Immunology, Tokyo, Japan) by manufacturer’s protocol.

### Rat model of SCI

Sprague-Dawley rats were anesthetized with 5% isoflurane until unconscious followed by 3% isoflurane during surgery. The T9–T10 laminoid and spinous processes were removed, and the spinal cord was exposed. Centered on the posterior median line, a 10 g hammer was used to hit the T9 segment of the spinal cord from a height of 25 mm to make an acute SCI model, and the incision was sutured layer by layer. Rats in the sham group underwent only laminotomy but did not undergo the heavy blow procedure. The rats were placed in cages for housing and observation. Cefazolin sodium was intraperitoneally injected twice daily (0.9%), and the rats were assisted to urinate artificially twice in the morning and evening.

### NSCs transplantation

Sprague-Dawley rats were randomly divided into four groups of twelve each as followings: (1) Sham group, (2) SCI group, (3) AAV2-5HRE-NSCs group, and (4) AAV2-5HRE-bFGF-NSCs group. NSCs were pre-transduced with 1 × 10^3^ MOI of AAV2-5HRE-bFGF or AAV2-5HRE virus particles for 48 h. Three days after the SCI was performed, the spinal cord was exposed, and 1 × 10^6^ NSCs re-suspended in 5 μl of PBS were injected into the SCI center (depth: 1 mm) of SCI rats by stereotaxic instrument and micro injection pump. Recovery from acute SCI was observed at ten time different points on days 1, 3, 7, 10, 14, 21, 28, 35, 42, and 60 after NSCs transplantation.

### Evaluation of functional recovery of SCI rats

The recovery of spinal cord function in rats was evaluated by BBB score, the oblique plate test, footprint, and video recording images. The methods were as follows: the rats were placed on a platform and allowed to move freely, and their hind limb walking, and limb movement were recorded. When it comes to inclined plate tests, one inclined plate which can adjust activity were preset on the desktop. The rats were placed on the 6 mm thick anti-skid pad, and the vertical axis of rat body and the long axis of inclined plate were perpendicular to each other. The angle between the desktop and inclined plate was slowly raised until rats could be on board for 5 s, and then recorded it. A 7.5 cm × 100 cm track was set up and covered with white paper. The track was covered with black plastic film to avoid light, so as to meet the adaptability of rats to the dark environment. The rats’ hind feet were marked with red dye and placed in the starting position of the track, allowing the animals to move from one end to the other. The footprint was observed to evaluate motor function. Video images were recorded and quantitatively analyzed as previously described^40^, including weight support, leg extensor spasms, the number of footsteps, and the posture of the foot.

### Hematoxylin–eosin (H&E) and nissl staining

Thoracotomy was performed on a randomly selected portion of SCI rats at the 60 days after cell transplantation. The heart was infused with 500 ml paraformaldehyde buffer solution, and the spinal cord was carefully separated. The spinal cord was placed in 2% paraformaldehyde for 6 h. H&E staining was performed and observed under an optical microscope. For nissl staining, the frozen sections were immersed in chloroform for 1 min and gradient alcohol for 1 min; respectively. After being rinsed twice with distilled water, they were dyed in tar purple dye for 30 min, and then dyed in a 37 °C box for 10 min. After being rinsed twice again by distilled water, 95% alcohol was used, followed by conventional dehydration, transparency, and tablet sealing treatment. Images were observed and photographed under an optical microscope.

### Western blot analysis

For the in vitro protein analyses, tissues were isolated and rapidly stored at −80 °C for western blot assays. For protein extraction, the tissues were homogenized in modified radioimmunoprecipitation assay (RIPA) buffer (50 mM Tris-HCl, 1% NP-40, 20 mM DTT, 150 mM NaCl, pH = 7.4) containing 10 µl/ml protease inhibitor cocktail (GE Health care Biosciences, PA, USA). The extract was then centrifuged at 12,000 rpm, and the supernatant was obtained for protein assay. Similarly, in vitro cultured NSCs were lysed in RIPA buffer (25 mM Tris-HCl, 150 mM NaCl, 1% Nonidet P-40, 1% sodium deoxycholate, and 0.1% SDS). The protein concentrations were quantified with bicinchoninic acid (BCA) reagents (Thermo, Rockford, IL, USA). Fifty microgram proteins per lane were used to load on a 11.5% gel and then transferred onto a polyvinylidene fluoride (PVDF) membrane (Bio-Rad, Hercules, CA, USA). The membrane was pre-incubated with 5% milk (Bio-Rad) in Tris-buffered saline (TBS) with 0.05% Tween 20 for 1 h, and added with each of the antibodies: anti-HIF-1α(1:300), anti-bFGF (1:300), anti-Beclin 1 (1:500), anti-LC3 II (1:1000), anti-P62 (1:1000), anti-NeuN (1:500), anti-GAP43 (1:500), anti-GFAP (1:500), anti-NF200 (1:500) and GAPDH (1:1000), and incubated at 4 °C overnight. The membranes were washed with TBS three times and added with HRP-conjugated secondary antibodies at room temperature for 1 h. Signals were detected by ChemiDoc™ XRS+ Imaging System (Bio-Rad), and band densities were measured with Multi Gauge Software of Science Lab 2006 (FUJIFILM Corporation, Tokyo, Japan), and quantified by Quantity One (version 4.5.2; Bio-Rad).

### Immunofluorescence staining

The harvested spinal cord was placed in 2% paraformaldehyde for 6–7 h, and then paraffin embedding, and sectioning were performed in a freezer slicer. The sections were dried at room temperature and then placed in a constant temperature incubator for 4 h. Dewaxing treatment was performed with xylene in the fume hood twice, each time for 10 min. After hydration with gradient alcohol for 5 min, samples were rinsed with PBS twice, 5 min each. H_2_O_2_ was added and incubated for 15 min, then washed with PBS three times, 5 min each. Cells were permeabilized with PBS plus 0.5% Triton X 100 (PBST) at room temperature for 20 min; rinsed with PBS three times, 5 min each round. The sections were sealed with 1% bovine serum albumin (BSA) at room temperature for 30 min. Primary antibody (1:500 dilution in 1% BSA) was added overnight at 4 °C, and incubation solution without antibody was used as a negative control. After restoring the temperature for 1 h, samples were rinsed with PBST three times, 5 min each. Corresponding secondary antibody conjugated with Alexa Fluor594 (red) or Alexa-Fluor488 (green) was added under dark conditions and incubated at 37 °C for 1 h, and then rinsed with PBST three times, 5 min each. DAPI was employed to stain the nuclei of cells. Fluorescent quenching agent was added, and the tablets were sealed and stored at 4 °C. Photographs were taken under a fluorescence microscope. Fluorescent images were taken from the regions of interests (ROI) in comparable matching anatomical regions between groups. Imagine J software was used to count ROI using six samples and then SPSS13 software to yield the statistical graph.

### RAPA and 3-MA treatment in SCI rats

RAPA and 3-MA were prepared as stock solutions in DMSO (25 mg/ml). Experimental rats were administrated with intraperitoneal injection with a dose of RAPA (0.5 mg/kg/day), and 3-MA (2.5 mg/kg/day), or vehicle right after SCI. All experimental rats received daily rehabilitation for 14 days. The BBB scores and the inclined plane test scores were recorded on day 3, 7, and 14.

### Statistical analysis

Data were expressed as mean ± standard deviation, and SPSS13.0 statistical software was used for *t* test, and *P* < 0.05 was used to indicate statistical difference.

## Results

### AAV2-mediated and HRE-directed expression of bFGF gene in NSCs

**Fig. 1:**
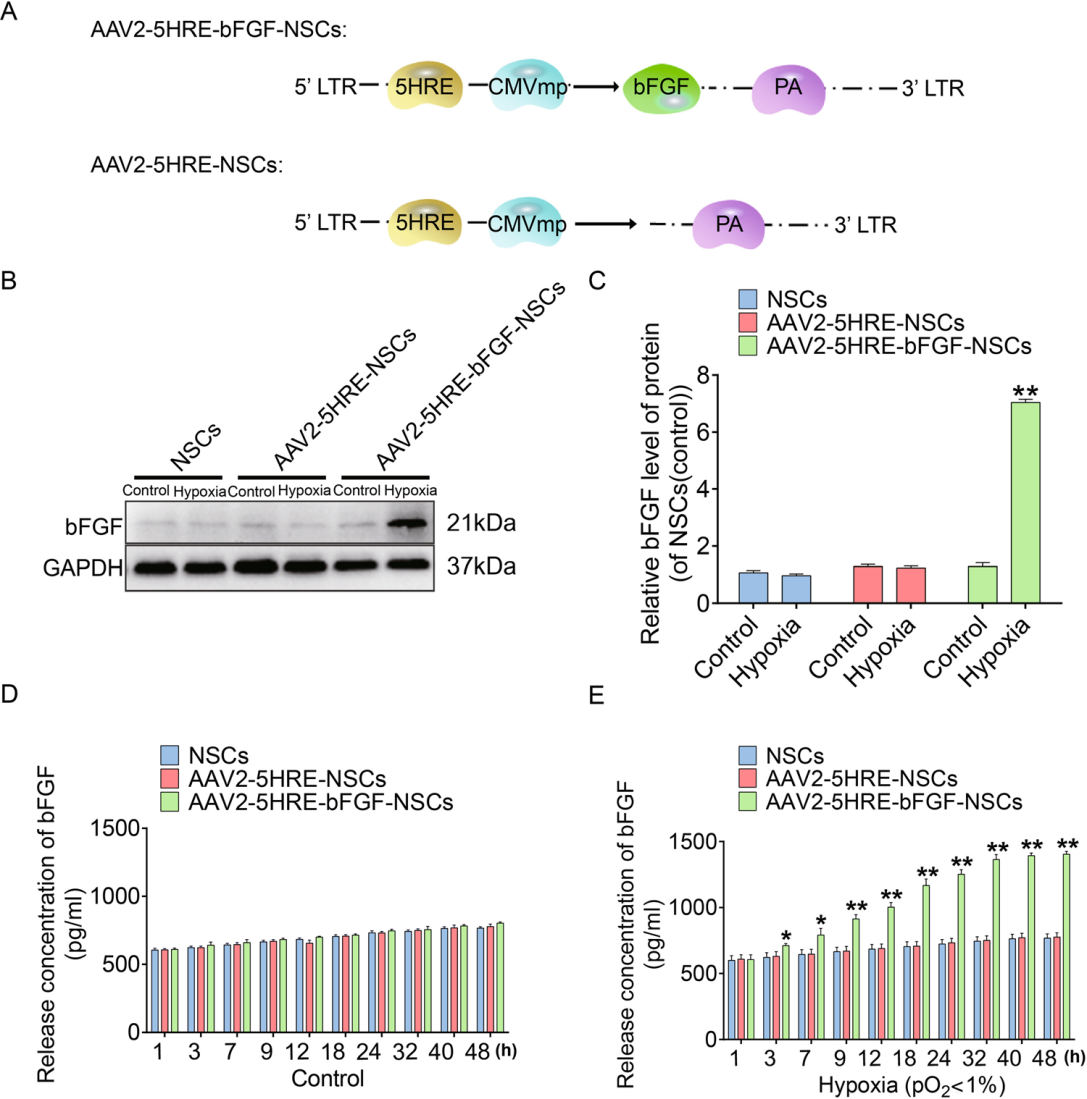
Preparation and characterization of the AAV2-5HRE-bFGF-NSCs. **A** Schematic diagram illustrating the construction of expression vectors AAV2-5HRE and AAV2-5HRE-bFGF. **B** Western blotting analyses of primary NSCs, AAV2-5HRE-NSCs, and AAV2-5HRE-bFGF-NSCs showing the expression of bFGF under normoxic or hypoxic conditions for 24 h. **C** The quantitative analyses of bFGF protein expression by western blot. **D** ELISA assay results showing bFGF secretion in primary NSCs, AAV2-5HRE-NSCs, and AAV2-5HRE-bFGF-NSCs under normoxic conditions. **E** ELISA assay results showing bFGF secretion of primary NSCs, AAV2-5HRE-NSCs, and AAV2-5HRE-bFGF-NSCs under hypoxic conditions. “*” and “**” represents *P* < 0.05 or *P* < 0.01 versus normoxia control. Data are the mean values ± SEM.

To examine AAV2-mediated bFGF expression in a hypoxia-inducible manner via the direct regulation of HRE, an AAV2-5HRE-bFGF construct was designed and generated (Fig. 1A), and then transfected into NSCs. By western blot analysis, bFGF protein expression in NSCs was induced under the hypoxia condition in vitro, but not in normoxia culture condition as compared with AAV2-5HRE blank vector control (Fig. 1B, C). Further, time course experiments using ELISA were conducted to examine bFGF protein expression, and the results showed that bFGF expression was induced under the hypoxia condition in a time course dependent manner but stayed in a residual level in normoxia culture condition, as compared with AAV2-5HRE blank vector control (Fig. 1D, E).

### AAV2-5HRE-bFGF-NSCs improve function recovery of SCI rats

**Fig. 2:**
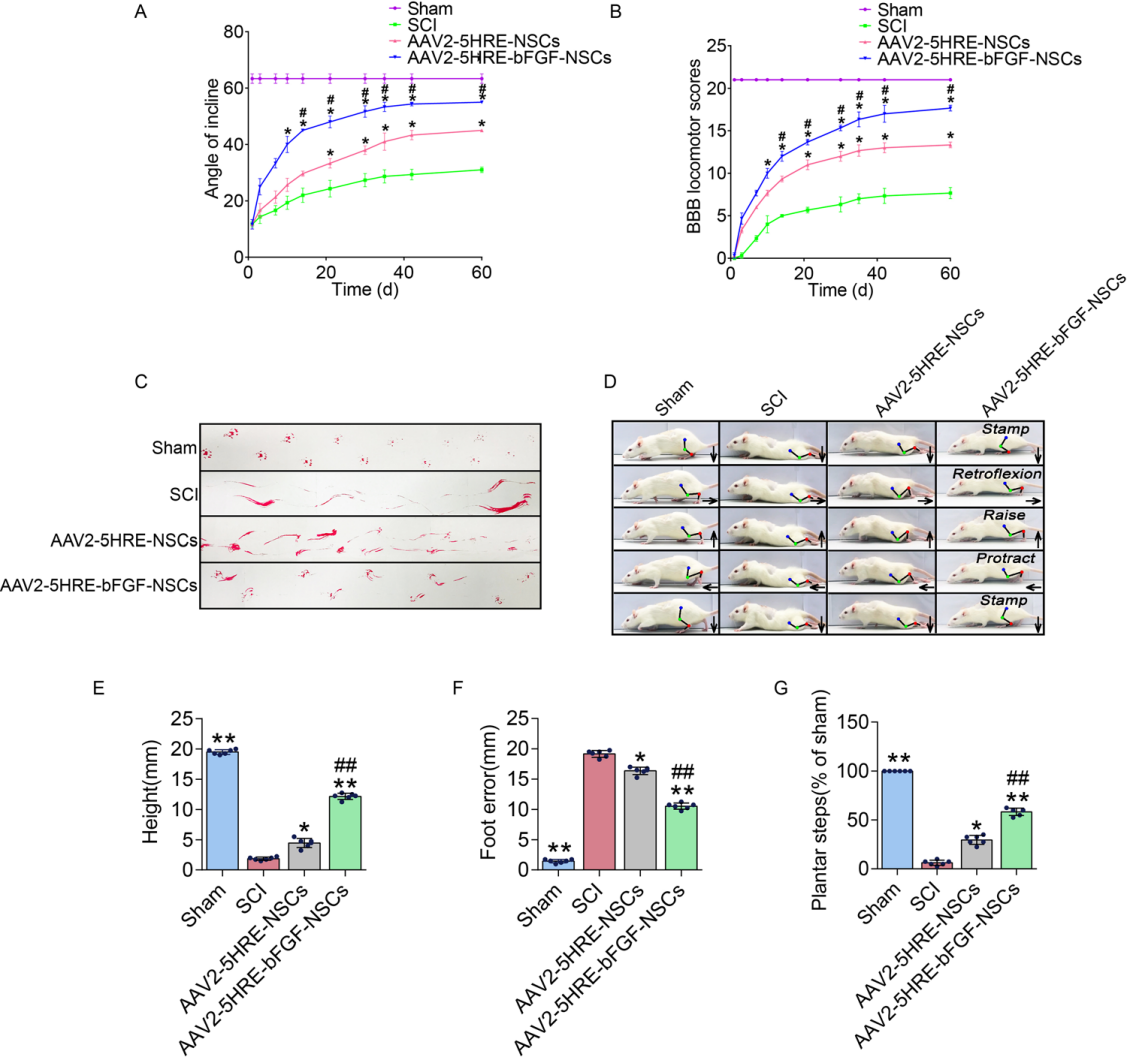
AAV2-5HRE-bFGF-NSCs improve the recovery of SCI rats. **A** The inclined plane test scores of sham, SCI group, AAV2-5HRE-NSCs group, and AAV2-5HRE-bFGF-NSCs group. **B** The BBB scales of sham, SCI group, AAV2-5HRE-NSCs group, and AAV2-5HRE-bFGF-NSCs group. “^#^” represents *P* < 0.05, AAV2-5HRE-NSCs group verse SCI group, “*” represents *P* < 0.05, AAV2-5HRE-bFGF-NSCs verse AAV2-5HRE-NSCs group. Data are the mean values ± SEM (*n* = 3). **C** Footprint analyses of sham, SCI group, AAV2-5HRE-NSCs group, and AAV2-5HRE-bFGF-NSCs group. **D** Video sequences of a rat walked 2 months after SCI were analyzed as previously described^40^, including weight support, leg extensor spasms, slow steps, and foot placement. Arrow denotes foot movement. Scale bar = 20 mm. **E** Weight support (quantified as the height of the trunk from the ground), **F** Foot error, and **G** Plantar step were presented. “**” represents *P* < 0.01, versus the sham group or SCI group, “^##^” represents *P* < 0.01, versus the AAV2-5HRE-NSCs group. Data are the mean values ± SEM (*n* = 6).

Having established AAV2-mediated and HRE-directed expression of bFGF gene in NSCs, which was named AAV2-5HRE-bFGF-NSCs. The AAV2-5HRE-bFGF-NSCs were then transplanted to the site of SCI rats 3 day after the induction of SCI. AAV2-5HRE-NSCs was used as a vehicle control. Functional recovery was evaluated on days 1, 3, 7, 10, 14, 21, 28, 35, 42, and 60 after NSCs transplantation. The oblique plate test results showed that AAV2-5HRE-bFGF-NSCs group scored higher than AAV2-5HRE-NSCs group and SCI groups in the (Fig. 2A). Consistently, BBB scales in the AAV2-5HRE-bFGF-NSCs group were also higher than AAV2-5HRE-NSCs group and SCI group (Fig. 2B). Further, in the footstep imprinting experiment, rats of sham group showed clear imprinting, orderly arrangement and along the same straight line. Rats in SCI group showed severe imprinting disorder with a wave shape and dragged movement. Rats in the AAV2-5HRE-NSCs group showed imprinting disorder mixed with some clear imprinting. In comparison, rats in the AAV2-5HRE-bFGF-NSCs group showed neat imprinting (Fig. 2C). These results indicate an improved functional recovery of AAV2-5HRE-bFGF-NSCs group as compared to SCI group and AAV2-5HRE-NSCs group.

In addition, the hind limb movement ability of rats in each group was also recorded and evaluated (Fig. 2D). As expected, in SCI group, the connection lines of hind limb joints were approximately horizontal and straight, and there was no obvious joint movement in lower limb paralysis. In the AAV2-5HRE-NSCs group and the AAV2-5HRE-bFGF-NSCs group, rats were able to continuously support body weight with the sole limb, and some of them were able to move the front and rear limbs together frequently. Notably, the AAV2-5HRE-bFGF-NSCs group displayed a wider range of joint motion compared with the AAV2-5HRE-NSCs group. Furthermore, in SCI group, rats had the lowest maximum height off the ground, the highest degree of footstep error, and the smallest foot paw movement range among all groups (Fig. 2D). Quantitative analyses revealed that the AAV2-5HRE-bFGF-NSCs group had a higher maximum height from the ground (Fig. 2E), a lower footstep error (Fig. 2F), and a larger range of foot movement (Fig. 2G) than the AAV2-5HRE-NSCs group and SCI group, indicative of an improvement of hind limb motor ability after the transplantation of AAV2-5HRE-bFGF-NSCs in SCI rats.

### AAV2-5HRE-bFGF-NSCs improve spinal cord anatomic appearance and histopathology in SCI rats

**Fig. 3:**
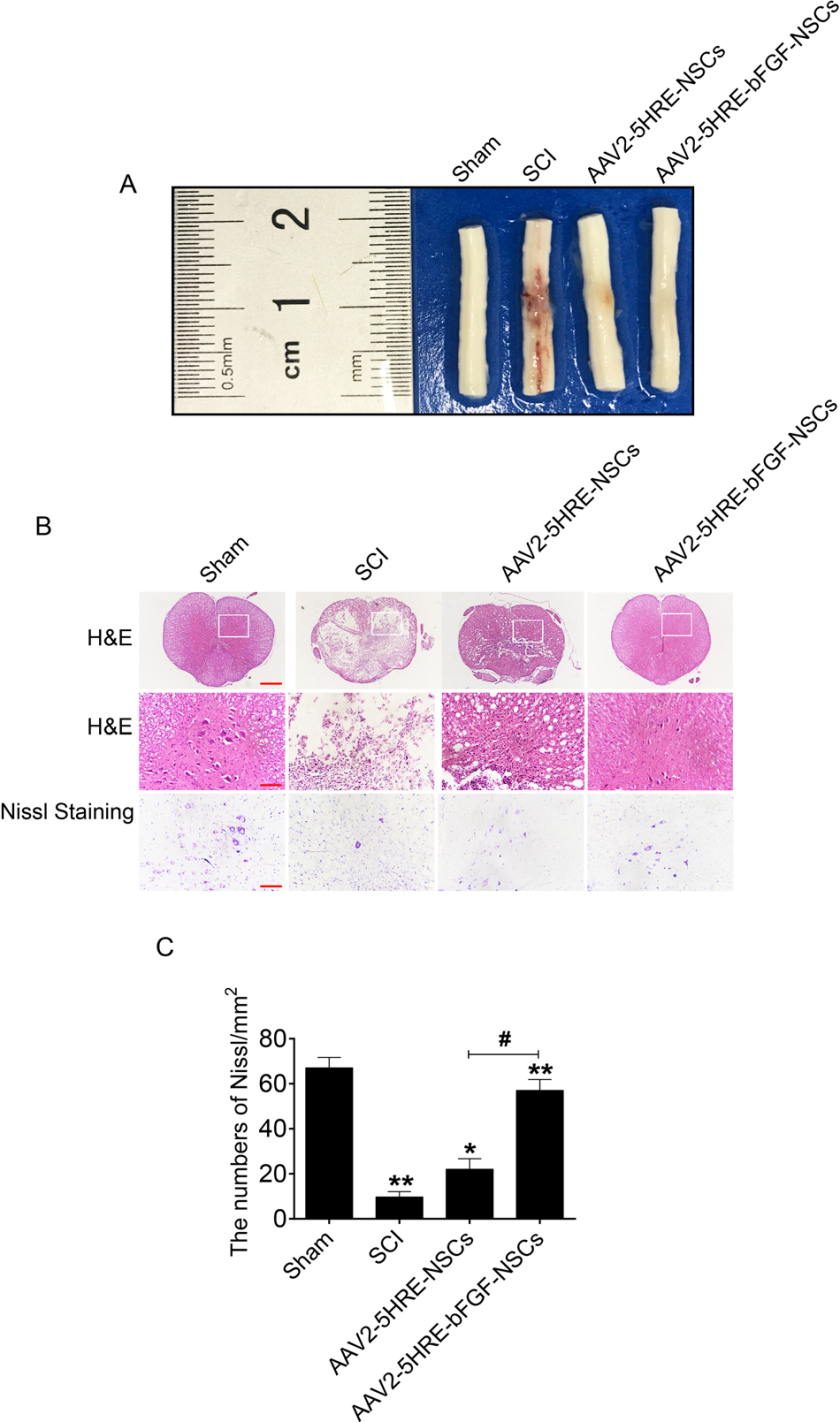
AAV2-5HRE-bFGF-NSCs improve the histopathology in SCI rats. **A** Anatomical observation of the spinal cord of rats. **B** H&E staining (cross-section) and nissel staining images for the sham group, SCI group, AAV2-5HRE-NSCs group and AAV2-5HRE-bFGF-NSCs group, scale bar = 500 µm. Representative regions with high power magnification were also presented, scale bar = 100 µm. Nissl staining of the different groups, scale bar = 100 µm. **C** Quantitative analysis of the nissl staining results. “**” represents *P* < 0.01 or “*” represents *P* < 0.05, versus sham group or SCI group. “^#^” represents *P* < 0.05, versus the AAV2-5HRE-NSCs group. Data are the mean values ± SEM (*n* = 6).

Global anatomical observation of the spinal cord of rats in each group showed that the SCI site of rats in SCI group became murkier and atrophic 60 days after SCI. The AAV2-5HRE-NSCs group showed a small range of murky regions with slight atrophy. In comparison, the AAV2-5HRE-bFGF-NSCs group showed little color change and atrophy (Fig. 3A).

Microscopic examination with H&E staining showed that after 60 days, SCI sites were atrophic in SCI group. Notably, central gray matter and peripheral white matter were necrotic with cystic cavities (Fig. 3B). Further, nissl staining showed that the number of neurons in SCI group decreased, and the number of nissl bodies almost disappeared compared with Sham group control (Fig. 3B, C). The AAV2-5HRE-NSCs group showed small and sporadic voids in the gray matter with no obvious necrosis, and nissl bodies were not significantly reduced (Fig. 3B, C). In comparison, the spinal cord of the AAV2-5HRE-bFGF-NSCs group was relatively full, and no obvious sacs were found. Further, histological structure was more intact with increased numbers of nissl positive neuron cells, when compared to SCI group and AAV2-5HRE-NSCs group (Fig. 3B, C).

### AAV2-5HRE-bFGF-NSCs show temporally increased expression of bFGF in SCI rats under the regulation of hypoxia

**Fig. 4:**
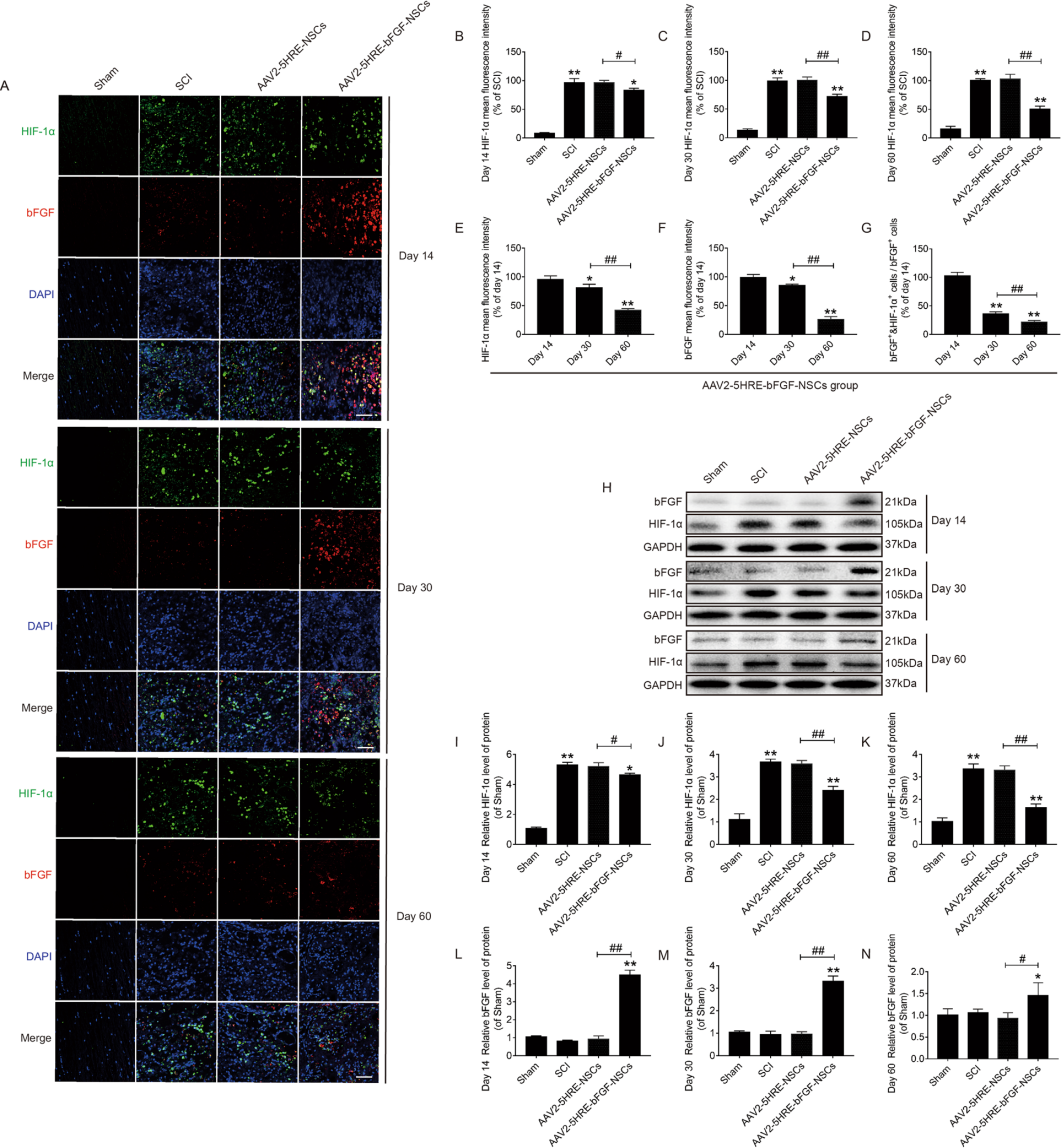
AAV2-5HRE-bFGF-NSCs show temporally increased expression of bFGF in SCI rats under the regulation of hypoxia. **A** Immunofluorescence staining of bFGF and HIF-1α in the sham group, SCI group, AAV2-5HRE-NSCs group and AAV-5HRE-bFGF-NSCs group in day 14, day 30 and day 60. The bright green signals indicate positive staining for bFGF. The bright red signals denote positive staining for HIF-1α. The nuclear is labeled by DAPI (blue), magnification was ×40. Scale bar = 50 µm. **B**–**D** Comparison of the fluorescence intensity of HIF-1α positive cells by immunofluorescence staining. **E** Comparison of the fluorescence intensity of HIF-1α positive cells by immunofluorescence staining in day 14, day 30, and day 60 of AAV-5HRE-bFGF-NSCs group. **F** Comparison of the fluorescence intensity of bFGF positive cells by immunofluorescence staining in day 14, day 30, and day 60 of AAV-5HRE-bFGF-NSCs group. **G** Comparison of the fluorescence intensity of bFGF^+^ and HIF-1α^+^ cells in bFGF^+^ cells by immunofluorescence staining in day 14, day 30, and day 60 of AAV-5HRE-bFGF-NSCs group. **H**–**N** Western blotting results of HIF-1α and bFGF protein expression levels in sham group, SCI group, AAV2-5HRE-NSCs group, and AAV2-5HRE-bFGF-NSCs group of day 14, day 30, and day 60. GAPDH was used as a reference. “**” represents *P* < 0.01 or “*” represents *P* < 0.05, versus SCI group in day 14. “^##^” represents *P* < 0.01 or “^#^” represents *P* < 0.05. Data are the mean values ± SEM (*n* = 3).

Immunofluorescence staining was performed to observe the expression of bFGF and HIF-1α, DNA fluorescence marker DAPI was used to stain the nucleus. It was revealed that HIF-1α positive rates were highest in the SCI group at three time points: day 14, day 30, and day 60. The HIF-1α positive rate in AAV2-5HRE-NSCs group was similar to that of the SCI group without statistical significance. The HIF-1α positive rate in AAV2-5HRE-bFGF-NSCs group was significantly lower than that of the SCI group (Fig. 4A–D). To further explore the reasons for the decreased HIF-1α positive rate, we conducted a combined analysis of HIF-1α and bFGF in the AAV2-5HRE-bFGF-NSCs group. We found that the HIF-1α positive rate was highest at 14 days, decreased over time, and lowest at 60 days (Fig. 4A, E). The positive rate of bFGF showed a trend similar to that of HIF-1α (Fig. 4A, F). At the same time, we found that the proportion of bFGF^+^ and HIF-1α^+^ cells in bFGF^+^ cells was decreased over time (Fig. 4A, G). These results indicate that expression of bFGF by AAV2-5HRE-bFGF-NSCs is accompanied by decreased HIF-1α expression, indicating that the expression of bFGF is regulated by hypoxia environment. Furthermore, we used WB analysis to verify the expression of HIF-1α at day 14, day 30, and day 60. It was found that the expression of HIF-1α in the SCI group was the highest. The expression of HIF-1αin AAV2-5HRE-NSCs group was similar to that of the SCI group without statistical significance. The expression of HIF-1α in the AAV2-5HRE-bFGF-NSCs group gradually decreased over time, which was close to the sham group and was significantly different from the SCI group (Fig. 4H–K). At day 14, the expression level of bFGF in the AAV2-5HRE-NSCs group was more than that of the SCI group with statistical significance, and the expression level of bFGF in the AAV2-5HRE-bFGF-NSCs group was more than that of the AAV2-5HRE-NSCs group with statistical significance. At 30 and 60 days, there was no statistical difference in bFGF expression between the AAV2-5HRE-NSCs group and the SCI group. The expression of bFGF in the AAV2-5HRE-bFGF-NSCs group was more than that of the SCI group and the AAV2-5HRE-NSCs group with statistical significance, but it gradually decreased over time (Fig. 4H, L–K). Collectively, these results indicate that the expression of bFGF is regulated by hypoxia environment.

### AAV2-5HRE-bFGF-NSCs increase the expression of neuron-specific NeuN and GAP43 proteins in SCI rats

**Fig. 5:**
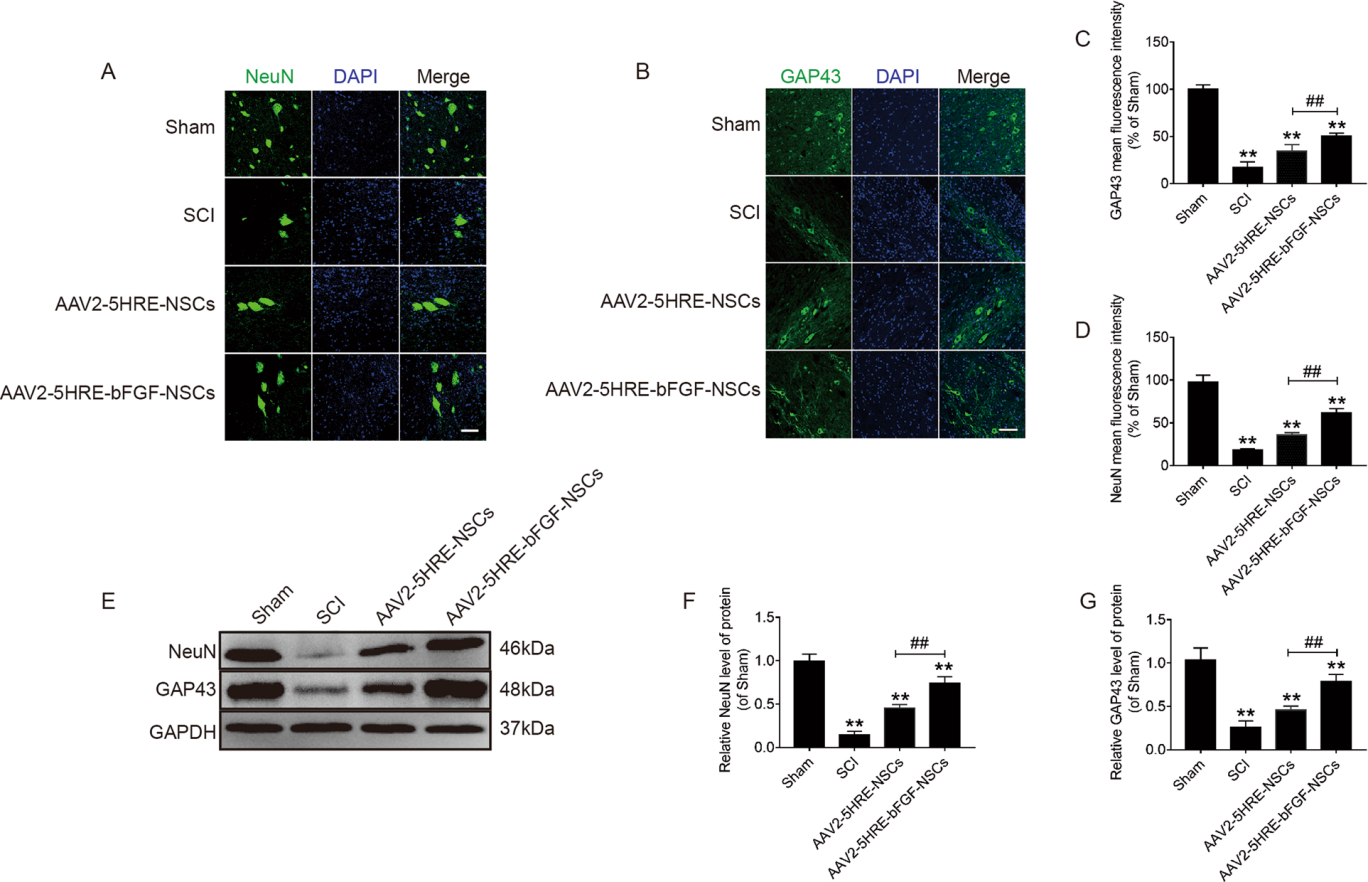
AAV2-5HRE-bFGF-NSCs upregulate the expression of NeuN and GAP43. **A** Immunofluorescence staining of NeuN in sham group, SCI group, AAV2-5HRE-NSCs group and AAV2-5HRE-bFGF-NSCs group. The bright green dots are considered as positive staining of neurons. The nuclear is labeled by DAPI (blue). Scale bar = 50 µm. **B** Immunofluorescence staining of GAP43 in sham group, SCI group, AAV2-5HRE-NSCs group, and AAV2-5HRE-bFGF-NSCs group. The label of nuclear is accomplished by DAPI (blue), the neurons with positive GAP43 signals are shown. Scale bar = 50 µm. **C**, **D** Quantitative analysis of NeuN and GAP43 fluorescence intensity results. **E**–**G** Western blotting results of NeuN and GAP43 protein expression levels in sham group, SCI group, AAV2-5HRE-NSCs group, and AAV2-5HRE-bFGF-NSCs group. GAPDH was used as a reference. “**” represents *P* < 0.01, versus the sham group or SCI group. “^##^” represents *P* < 0.01, versus the AAV2-5HRE-NSCs group. Data are the mean values ± SEM (*n* = 3).

NeuN (also named RNA binding protein fox-1 homolog 3 or Rbfox3) and neuromodulin GAP43 proteins, which regulate neuron growth and exon elongation were used to evaluate the outcomes of neural regeneration. Using immunofluorescence staining, NeuN and GAP43 showed more positive green fluorescence signals in spinal cord tissues in the AAV2-5HRE-bFGF-NSCs group compared with the AAV2-5HRE-NSCs group and SCI group (Fig. 5A–D). Further, using western blot analysis, the protein expression of NeuN and GAP43 in AAV2-5HRE-NSCs group and AAV2-5HRE-bFGF-NSCs was higher than that in SCI group. Notably, the protein expression level of AAV2-5HRE-bFGF-NSCs was significantly higher than that of AAV2-5HRE-NSCs (Fig. 5E–G). These results indicated the neuroprotective and neuron modulation effects of AAV2-5HRE-bFGF-NSCs in SCI is in line with the upregulation of NeuN and GAP43 proteins.

### AAV2-5HRE-bFGF-NSCs increase the expression of neurofilament protein NF200 and attenuate the expression of GFAP in SCI rats

**Fig. 6:**
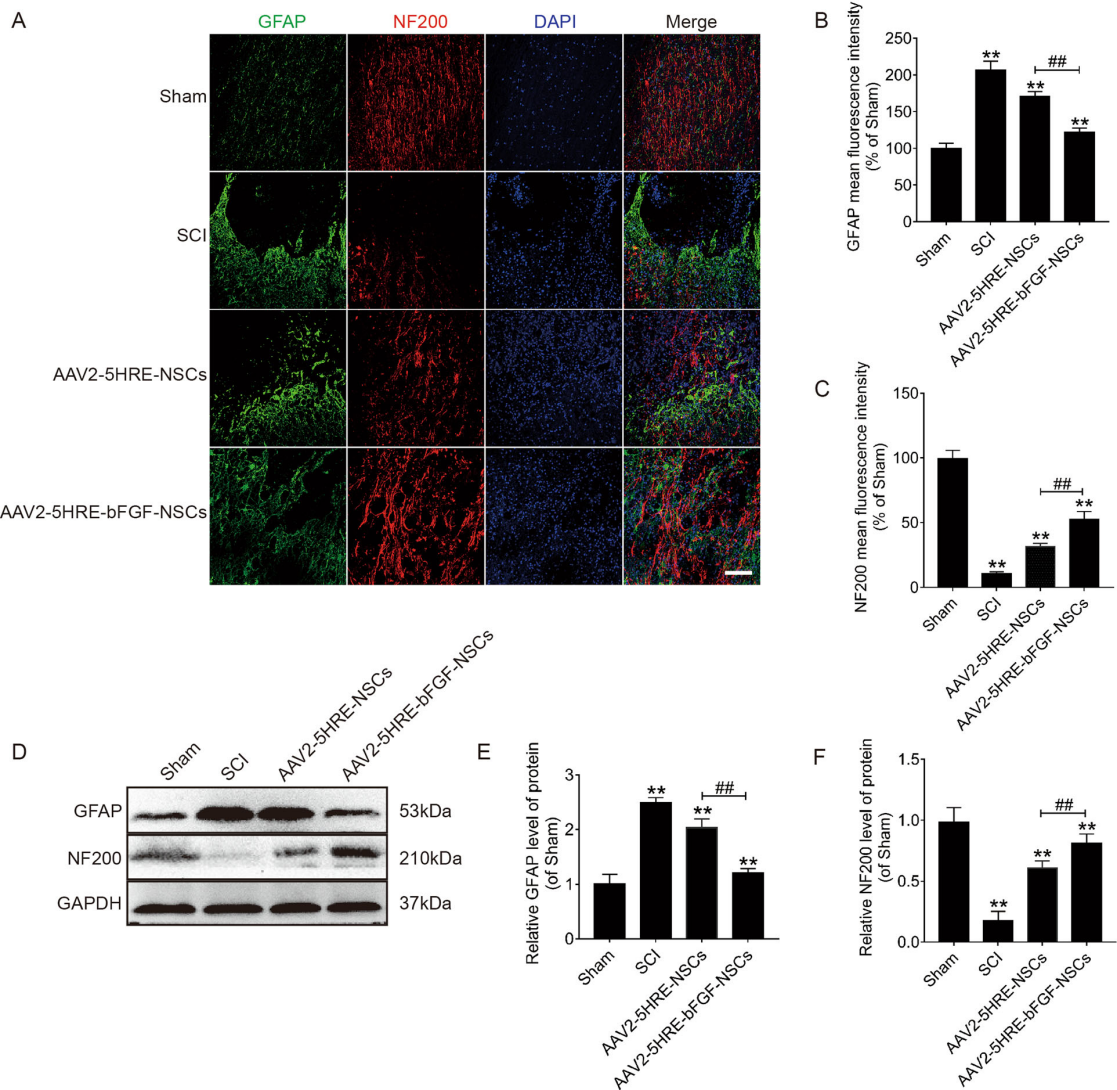
AAV2-5HRE-bFGF-NSCs increase the expression of neurofilament protein NF200 and attenuate the expression of GFAP in SCI rats. **A** Immunofluorescence staining of GFAP and NF200 in sham group, SCI group, AAV2-5HRE-NSCs group and AAV2-5HRE-bFGF-NSCs group. The bright green dots are considered as GFAP positive staining. The bright red dots are considered as NF200 positive staining. The nuclear is labeled by DAPI (blue). Scale bar = 100 µm. **B**, **C** Quantitative analysis of GFAP and NF200 fluorescence intensity results. **D**–**F** Western blotting images and quantitative analyses of GFAP and NF200 protein expression levels. GAPDH was used as a protein loading reference. “**” represents *P* < 0.01, versus the sham group or SCI group. “^##^” represents *P* < 0.01, versus the AAV2-5HRE-NSCs group. Data are the mean values ± SEM (*n* = 3).

Next, the expression of glial fibrillary acidic protein (GFAP), which is involved in glial scarring in SCI was examined. By confocal analysis, it was revealed that GFAP expression signals was lower in the AAV2-5HRE-bFGF-NSCs group, compared to in SCI group and AAV2-5HRE-NSCs group (Fig. 6A, B). Interestingly, GFAP expression cells in SCI group were restricted in the injury site within the scar forming line, whereas GFAP expression cells were more widely distributed in AAV2-5HRE-bFGF-NSCs group (Fig. 6A). Similarly, using Western blot analysis, the protein expression of GFAP was lower in AAV2-5HRE-bFGF-NSCs group than in SCI group and AAV2-5HRE-NSCs group (Fig. 6E–G).

Further, the expression of NF200, a neurofilament protein involved in axonal sprouting was investigated. Confocal analysis showed that NF200 expression in AAV2-5HRE-bFGF-NSCs group was higher compared to in SCI group and AAV2-5HRE-NSCs group (Fig. 6A, C). Interestingly, NF200 expression was present as tree-branching contour in AAV2-5HRE-bFGF-NSCs group, which resembles those of the sham group, indicative of the presence of axonal sprouting (Fig. 6A). Consistently, using Western blot analysis, NF200 expression was higher in AAV2-5HRE-bFGF-NSCs group than in SCI group and AAV2-5HRE-NSCs group (Fig. 6E–G). Collectively, these experimental data suggest that there was an improved outcome in glial scar inhibition, axon regeneration expanding over the scar boundary, and growth of axon regeneration in AAV2-5HRE-bFGF-NSCs group.

### AAV2-5HRE-NGF-NSCs show reduced expression of LC3-II and Beclin 1, and augmented expression of P62 proteins in SCI rats

**Fig. 7:**
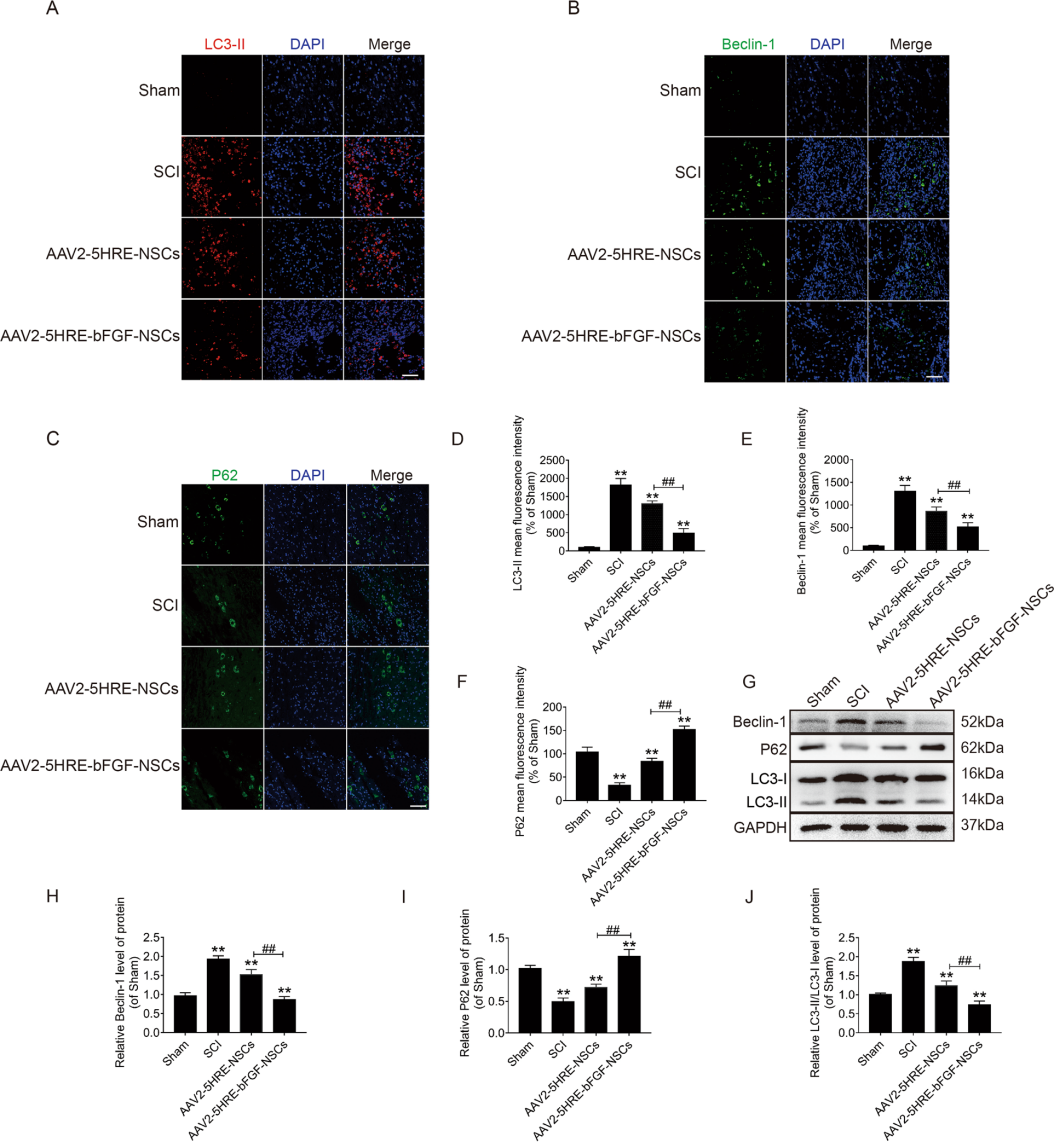
AAV2-5HRE-bFGF-NSCs show reduced expression of LC3-II and Beclin 1, and augmented expression of P62 proteins in SCI rats. **A**, **B**, **C** Immunofluorescence staining of LC3-II, Beclin 1 and P62 in sham group, SCI group, AAV2-5HRE-NSCs group, and AAV2-5HRE-bFGF-NSCs group. The nuclear is stained with DAPI (blue). Scale bar = 50 µm. **D**, **E**, **F** Quantitative analyses of fluorescence intensity results. **G** Western blotting results of Beclin 1, P62, and LC-3 expression levels. GAPDH was used as an internal reference. Quantitative analyses of Western blots of Beclin 1 (**H**), P62 (**I**), LC3 (**J**) are presented. “**” represents *P* < 0.01, versus the sham group or SCI group. “^##^” represents *P* < 0.01, versus the AAV2-5HRE-NSCs group. Data are the mean values ± SEM (*n* = 3).

To determine whether the mechanism of AAV2-5HRE-NGF-NSCs’s action was related to the modulation of autophagy pathway, the autophagy-associated proteins were examined by immunofluorescence staining. It was revealed that the number of both LC3-II and Beclin 1 positive cells was decreased, whereas the number of P62 positive cells was increased in AAV2-5HRE-bFGF-NSCs group as compared with and SCI group and AAV2-5HRE-NSCs group (Fig. 7A–F). Consistently, western blot analysis showed that LC3-II and Beclin 1 protein levels were decreased, while P62 protein level was augmented in AAV2-5HRE-bFGF-NSCs group as compared with SCI group and AAV2-5HRE-NSCs group (Fig. 7G–J). Together these data suggest that effects of AAV2-5HRE-NGF-NSCs on the functional recovery of SCI rats might be attributed to the inhibition of SCI-induced autophagy by AAV2-5HRE-NGF-NSCs.

To further attest if AAV2-5HRE-bFGF-NSCs have inhibitory effects on autophagy, NSCs were treated with RAPA, an agonist of autophagy, and showed that LC3-II was upregulated in the presence of RAPA. In AAV2-5HRE-bFGF-NSCs + RAPA group, the fluorescence intensity of LC3-II was lower than that of NSCs + RAPA group and AAV2-5HRE-NSCs + RAPA group (Supplementary Fig. 1A). Consistently, 3-MA, an inhibitor of autophagy was shown to attenuate the effect of RAPA. The fluorescence intensity of LC3-II was reduced when cells in each group are treated with 3-MA after RAPA stimulation (Supplementary Fig. 1B). Similarly, by Western blot analyses, LC3-II and Beclin 1 protein expression were decreased, while P62 protein expression was increased in AAV2-5HRE-bFGF-NSCs + RAPA group when compared to NSCs + RAPA group and AAV2-5HRE-NSCs + RAPA group (Supplementary Fig. 1C–F), which further attested that AAV2-5HRE-bFGF-NSCs can attenuate RAPA-induced cell autophagy. Finally, to evaluate if inhibiting autophagy can facilitate the functional recovery of SCI, the effects of RAPA and RAPA + 3-MA treatments on SCI rats were investigated. It was revealed that the BBB scales and the inclined plate test scores of RAPA + 3-MA treated group were higher than those of SCI group and SCI + RAPA group (Supplementary Fig. 1G, H). These data suggest that induction of autophagy by RAPA exacerbated, whereas attenuation of autophagy by 3-MA improved the functional recovery of SCI in rats. These results are in line with the therapeutic effect of AAV2-5HRE-bFGF-NSCs on SCI in rats (Fig. 2), which is attributed at least in part to its inhibitory effect on SCI-induced autophagy (Fig. 7).

## Discussion

In recent years, the socioeconomic and medical costs of SCI have continued to increase worldwide, placing an immeasurable burden on individuals and health care systems^41,42^. Embryonic NSCs have extended survival ability and low immunogenicity, and transplantation of NSCs has been proposed for the treatment of SCI^43–45^. At cellular level, NSCs can replace damaged neurons, oligodendrocytes, and astrocytes, and promote axon regeneration and myelin sheath reconstruction^46–48^. At molecular level, neurotrophic factors released by NSCs with the ability of promoting regeneration can help restore the function of the damaged spinal cord^49^. In addition, non-neural tissue near the site of SCI can also be repaired^50^. However, studies have found that NSCs often fail to survive and function in SCI^51^. The reason is that after SCI, the damaged tissues release excitatory neurotransmitters, oxygen free radicals and other toxic substances, forming a local microenvironment unfavorable to the survival of transplanted NSCs, which could lead to irreversible cell death or apoptosis of transplanted cells^52,53^. The high sensitivity and lack of adaptable regulation of NSCs to alterations in ambient oxygen level have been a major hurdle to overcome in the stem cell therapy of SCI.

Studies have shown that autophagic death of neurons and glial cells plays a role in SCI-induced neuronal damage, and inhibition of autophagy may serve as a therapeutic strategy for SCI^54^. It has been suggested that autophage signaling plays a direct role in inducing cell death in neuronal injury, and exceeding autophagy could result in cell death^55–58^. P62 is one of the marker proteins that are associated with autophagy activity, and its levels indirectly reflect the degree of autophagy body clearance. During autophagy, P62, as a ubiquitin binding protein mediates ubiquitinated proteins, and forms protein complex located on the inner membrane of autophagy body, leading to autophagy-mediated P62 protein degradation in lysozyme^59^. In contrast, when autophagy activity is destabilized, P62 protein will continuously accumulate in the cytoplasm. On the other hand, the Beclin 1 and LC3-II protein levels are raised during autophagy^60,61^. At the day of 60 after SCI, we found that AAV2-5HRE-bFGF-NSCs group showed an increase of P62 expression and a decrease of Beclin 1 and LC3-II expression compared to SCI group and AAV2-5HRE-NSCs group, indicating that autophagy is inhibited, consistent with the improved functional recovery of SCI by AAV2-5HRE-bFGF-NSCs.

bFGF is a key neurotrophic factor that promotes nerve growth and survival^30,62^. Previous studies have shown that bFGF can attenuate a series of secondary pathological changes caused by nerve trauma by reducing oxygen free radicals, antagonizing calcium overload and decreasing the cytotoxicity of nitric oxide^63^. Many scholars have found that bFGF could be temporarily expressed in some parts of spinal cord, which is consistent with the observed growth of neurons and axons in the spinal cord^64,65^. However, its effect is highly time-sensitive, and easily affected by the surrounding environment, and thus is lack of a stable and lasting impact on SCI repair^66^.

The purpose of this study is to use NSCs modified by the oxygen-regulated bFGF gene to overcome the ischemic and hypoxic microenvironment to promote the growth of neurons and improve the repair efficiency of SCI. To generate NSCs transfected with oxygen-regulated bFGF gene from embryonic spinal cord, a synthetic 5HRE was used to construct an oxygen-regulated recombinant vector. To enable the function of NSCs after gene transfection is safe, we chose AAV2 as the carrier. AAV2 is the simplest single-stranded DNA-deficient virus which has been widely used in preclinical research and in clinical trials^37,38^. The results of hypoxic induced expression of bFGF in NSCs via AAV2 showed that this method enables AAV2-5HRE-bFGF-NSCs to temporally express bFGF in an oxygen-regulated manner in SCI rats. The use of AAV2 is advantageous for future human clinical trials than lentivirus-based vector, considering that lentivirus-mediated expression of bFGF has been shown to improve the functional recovery of SCI rats^39^.

In conclusion, we found that the application of AAV2-5HRE-bFGF-NSCs showed therapeutic effects on the functional recovery of SCI in rats. AAV2-5HRE-bFGF-NSCs also increase protein expression of neuron-specific proteins and reduce SCI-induced glial scar formation and cell autophagy. Collectively, this study may provide valuable preclinical data for potential application of AAV2-5HRE-bFGF-NSCs to the clinic to treat SCI.

## Supplementary information

Sfigure 1

Supplementary figure legends

## Data Availability

The data used to support the findings of this study are available from the corresponding author upon request.
