# Supplementary figures and images for: AAV2-mediated and hypoxia response element-directed expression of bFGF in neural stem cells showed therapeutic effects on spinal cord injury in rats
Source: Cell Death Dis. 2021 Mar 15;12(3):274. doi: 10.1038/s41419-021-03546-6 (PMC7960741; doi:10.1038/s41419-021-03546-6)

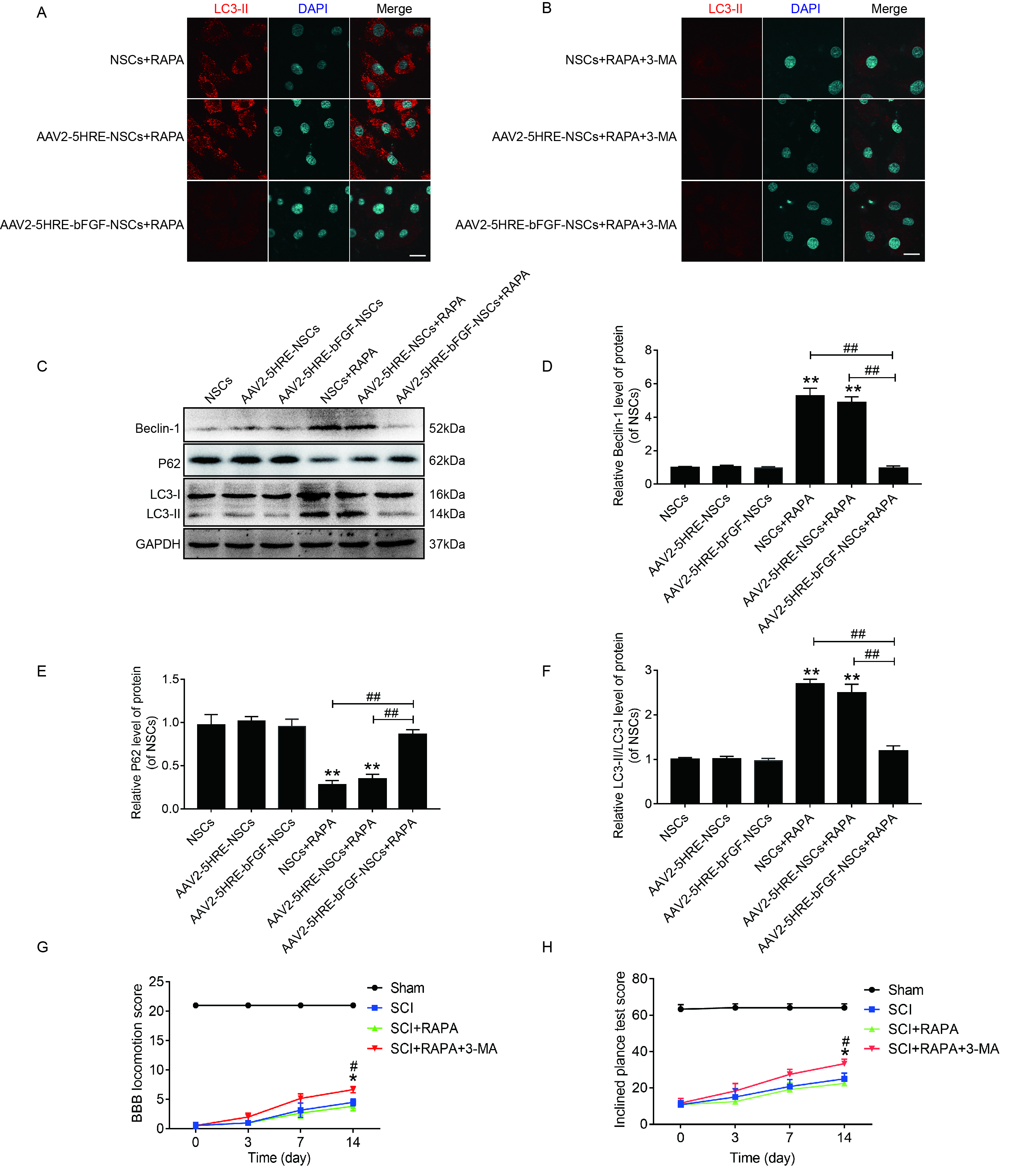

Supplement: Supplementary file 1 — Sfigure 1 [file 41419_2021_3546_MOESM1_ESM.tif]
