# Supplementary material for: AAV2-mediated and hypoxia response element-directed expression of bFGF in neural stem cells showed therapeutic effects on spinal cord injury in rats
Source: Cell Death Dis. 2021 Mar 15;12(3):274. doi: 10.1038/s41419-021-03546-6 (PMC7960741; doi:10.1038/s41419-021-03546-6)
Supplement: Supplementary file 2 — Supplementary figure legends [file 41419_2021_3546_MOESM2_ESM.docx]

**Supplement Figure 1.** **AAV2-5HRE-bFGF-NSCs attenuate RAPA-induced autophagy.**

**(A-B)** Immunofluorescence staining of LC3-II staining in NSCs, AAV2-5HRE-NSCs and AAV2-5HRE-bFGF-NSCs after RAPA treatment, and RAPA plus 3-MA treatment. The bright red dots are considered as LC3-II positive staining. The nuclear is labeled by DAPI (blue). Scale bar = 50μm. **(C)** Western blotting images of Beclin 1, P62 and LC3-II protein expression levels. GAPDH was used as an internal reference. **(D-F)** Quantitative analyses of Beclin 1, P62 and LC3-II protein expression levels of Western blotting results. Data are the mean values ± SEM (n=3). **(G)** The BBB scores of sham group, SCI group, SCI+RAPA group and SCI+RAPA+3-MA group. **(H)** The inclined plane test scores of sham group, SCI group, SCI+RAPA group and SCI+RAPA+3-MA group. Data are the mean values ± SEM ( n=6). “*” represents P < 0.05, versus the SCI group. “#” represents P < 0.05, versus the SCI+RAPA group.
